# RCT of Working Memory Training in ADHD: Long-Term Near-Transfer Effects

**DOI:** 10.1371/journal.pone.0080561

**Published:** 2013-12-09

**Authors:** Kjell Tore Hovik, Brit-Kari Saunes, Anne Kristine Aarlien, Jens Egeland

**Affiliations:** 1 Division of Mental Health and Addiction, Vestfold Hospital Trust, Tønsberg, Norway; 2 Department of Psychology, University of Oslo, Oslo, Norway; 3 Division of Child and Adolescent Psychiatry, Telemark Hospital Trust, Skien, Norway; Langeveld Institute, Utrecht University, The Netherlands

## Abstract

**Objective:**

The aim of the study is to evaluate the long-term near-transfer effects of computerized working memory (WM) training on standard WM tasks in children with Attention-Deficit/Hyperactivity Disorder (ADHD).

**Method:**

Sixty-seven children aged 10–12 years in Vestfold/Telemark counties (Norway) diagnosed with F90.0 Hyperkinetic disorder (ICD-10) were randomly assigned to training or control group. The training group participated in a 25-day training program at school, while the control group received treatment-as-usual. Participants were tested one week before intervention, immediately after and eight months later. Based on a component analysis, six measures of WM were grouped into composites representing Visual, Auditory and Manipulation WM.

**Results:**

The training group had significant long-term differential gains compared to the control group on all outcome measures. Performance gains for the training group were significantly higher in the visual domain than in the auditory domain. The differential gain in Manipulation WM persisted after controlling for an increase in simple storage capacity.

**Conclusion:**

Systematic training resulted in a long-term positive gain in performance on similar tasks, indicating the viability of training interventions for children with ADHD. The results provide evidence for both domain-general and domain-specific models. Far-transfer effects were not investigated in this article.

Trial Registration: Controlled-Trials.com ISRCTN19133620

## Introduction

Studies consistently show working memory (WM) to be impaired in children with Attention-Deficit/Hyperactivity Disorder (ADHD) [1]. WM processes mediate a range of cognitive skills contributing to lower academic performance [2] and poorer adaptive functioning in major life activities for young adults with ADHD [3]. Proponents of computerized WM training advocate the use of training programs to treat the cognitive deficits associated with ADHD, and point to studies suggesting that children with ADHD can improve their WM by practicing on adaptive computer-based programs [4], [5]. However, the effectiveness of WM training has been seriously questioned recently [6], [7], and the lack of a theoretical consensus on the construct working memory [8], [9] complicates the interpretation of results from intervention studies.

Computerized training studies involving children with ADHD commonly assess WM by administering tasks measuring WM capacity [7], [10]. Most of these training studies presume a two-component view of WM, consisting of 1) transient storage of information, and 2) manipulation of information remembered transiently [4], [5], [11], [12]. A distinction between visual and auditory modalities is also common in these studies [5], [11]–[13]. For all practical purposes, the storage and manipulation components of WM are equivalent to the specific temporary memory systems and separate resources (slave systems) supporting processing and multitask coordination (Central Executive) in the *Multicomponent Model* proposed by Baddeley [14], [15]. The model divides the role of attention in controlling action into two distinct mechanisms; one which is automatic and the other that is attentionally demanding, for example, when encountering a novel problem [16]. Several brain areas and networks are likely involved in WM processes, but considerable evidence suggests that activation in the Dorsolateral Prefrontal Cortex correlates with increasing manipulation load on WM tasks [17], [18]. It is precisely the manipulation aspect of WM that is thought to be impaired in children with ADHD as a result of frontostriatal dysfunction [1] and/or energetic dysfunction in pre-frontal systems [19], [20].

The brain's plasticity has been documented even in adults [21]–[23], and preadolescents are undergoing dynamic changes in grey and white matter substance as a natural part of their development [24]. Changes may be easily precipitated in young brains already undergoing dynamic change, but a central question is whether the effect of the change through training applies to similar (near) or dissimilar (far) tasks. Although a taxonomy of learning transfer has yet to be accurately described [25], *near-transfer* of learning refers to a gain in skill within the same training context; *far-transfer*, on the other hand, refers to the functional transfer of learning from one training context to anothe`r. A clear distinction should therefore be made between a gain in task-related performance and a gain in ability. Similar to any athlete training individual components of his or her sport, however, the precise relationship of how an improvement in a specific skill contributes to an overall ability is not always straight-forward. The indirect effects on measures of WM are published elsewhere [26]. The current examination of the effects of a computerized WM training program focuses exclusively on the near-transfer effects of such training. The theoretical separation of learning transfer effects is relevant to the current debate about the WM construct that revolves around whether WM resources are separable into pools of domain-specific resources (e.g. visuospatial and auditory WM) [27]–[29] or are better accounted for in a domain-general model, in which a centralized component (e.g. The Central Executive) operates on information in short-term, domain-specific storage components [14], [30]. The pattern of near-transfer effects from training intervention studies could contribute to the theoretical debate by identifying the impact of specific training on domain-specific versus domain-general components of WM.

Most PC-based training studies on children with ADHD administer a battery of tests and report gains on particular WM measures. Strong criticism has been raised of the single-test approach to assessing cognitive functions [10]. Due to the diffuse nature of the WM construct, single tests alone should not be interpreted as an increase in function. Multiple tasks tapping the same construct but differing in other respects (e.g. design, modality) provide a more robust outcome measure [10] and a more powerful predictive tool [31]. In response to this criticism, the current study uses composite measures grouped empirically through a component analysis to assess the impact of training on the WM construct.

The subjects in the study were tested with six tasks assumed to measure aspects of WM. In response to the criticism leveled at using single task scores, the six measures were grouped in composite scores based on a principal component analysis. First, when comparing the performance by the training group to the control group, we expect that there should be a significant gain by the training group compared to the control group on all composites as a consequence of having trained systematically on similar tasks over a five-week period. Second, we expect any improvement to be most evident in composites relating to the visual domain, as the exercises in the WM training program are presented predominantly visually (only 4 of 13 depend on auditory input). Third, if the component analysis yields a manipulation composite that is differentially improved by training, we need to investigate whether it represents an actual increase in the manipulation system or is merely an artifact of increased storage capacity reducing the cognitive load on the controlled manipulation tasks. In order to test for this, we will control for an increase in simple storage capacity. An increase in manipulation beyond a gain in simple storage capacity may be an indication of a genuine improvement in the executive component of WM. The persistence of the predicted gains for the training group compared to control group over the long term is unclear due to the lack of published studies showing lasting effects from training on similar WM measures [6].

## Methods

### Participants

**Figure 1:**
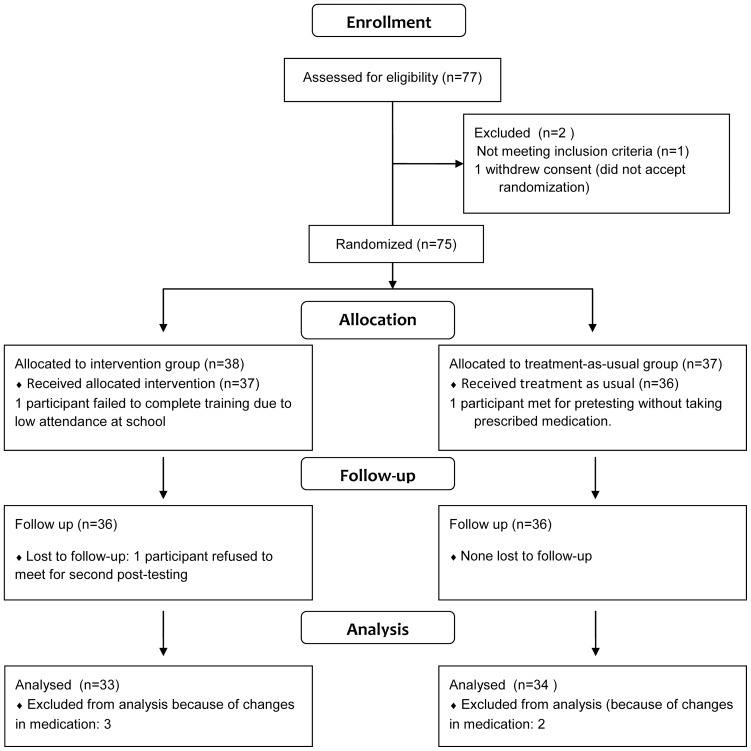
The diagram specifies the flow of participants through the enrollment, allocation, follow-up and analysis phases of the RCT study.

The flow of participants in the study is presented in Figure 1. The protocol for this trial and supporting Consort checklist are available as supporting information, see Checklist S1 and Protocol S1 and S2.

Candidates for the study were recruited by their therapist. They had to be 10–12 years of age and previously diagnosed at specialist outpatient clinics with F90.0 Hyperkinetic disorder (ICD-10) (WHO, 1992), equivalent to the DSM-IV diagnosis of ADHD combined type, and in treatment for ADHD within the Departments for Child and Adolescent Psychiatry in Vestfold or Telemark Hospital Trusts, Norway. Exclusion criteria were IQ below 70 (WISC-III or WISC-IV), or a comorbid diagnosis of Pervasive Developmental Disorder, Tourette's Syndrome, Conduct Disorder or evidence of psychosis or epilepsy. Only one candidate was excluded based on the exclusion criteria.

Seventy two of the original 77 children recruited to participate in the study completed the study. Of the 72 children completing the study, five children were omitted from the analyses presented here due to changes in medication during the project period.

Of the 67 children included in the presented analyses, forty-one of the participants were medicated with methylphenidate (MPH) and five with atomoxetine in the same dosage throughout the study. One patient was medicated with risperidone as well. Twenty-one participants did not use medication at the time of inclusion. Importantly, no participants included in the analyses started up on medication or changed dosage after the Pre-test, i.e. no risk of medication effects confounding training effects. All children 12 years of age and more and their parents signed written consent forms prior to participation.

Demographic information is presented in Table 1. There were no significant differences between the groups with regard to sex, age, medication status, IQ or education level of parents. Sixty percent of all participants received special education at school in the form of more individualized, structured instruction by a designated resource person. Thirty-five percent came from homes with a single parent/caregiver. There were no significant differences between the intervention and control groups on any of the demographic measures. The ratio of boys to girls in the current study is 3∶1, which is the same approximate gender ratio found in international epidemiological studies for ADHD (Gershon, 2002). The IQ level of participants is slightly under the average level for non-clinical groups, but at a similar level for ADHD cohorts [32], [33].

**Table 1:** Demographic and clinical characteristics: means and standard deviations.

Variable	Training group (*n* = 33)	Control group (*n* = 34)	*p*
Age in year (SD)	10.5 (0.7)	10.3 (0.8)	*ns*
Sex (male/female)	24/9	25/9	*ns*
Medication (yes/no)	23/10	24/11	*ns*
WISC-IV VC^1^	96 (12)	99 (11)	*ns*
WISC-IV PO^2^	95 (16)	99 (16)	*ns*
Full scale IQ	92 (12)	96 (12)	*ns*

1 ***Verbal Comprehension index score,*** ^2^ ***Perceptual Organization index score.***

### Trial design

The invitation to participate in the study and consent form emphasized that the study was a randomized controlled trial, and that half of the participants would be drawn at random to take part in the training. Participants in the control group were offered the chance to participate in the training program immediately after conclusion of the study. During the study period, all of the participants continued with treatment-as-usual, which included regular follow-up by a health professional at the local specialized youth health services facility and the same special education classes at school that they had participated in before the start of the study. No difference was made between the groups in their already existing treatment or educational programs, except for the addition of the WM training program for the children randomly assigned to the training group.

The experimental design included a pretest immediately before the start of the training period, a post-test at the conclusion of the training period and follow-up testing. At each stage of testing, the children in training and control groups were tested in the same time period during the day in a randomly assigned sequence. The original plans called for follow-up testing to take place six months after conclusion of the training period, but due to unforeseen delays in completing the training program due to a flu epidemic, the actual follow-up testing took place eight months after conclusion of the training period. Participants meeting the inclusion criteria were matched as to gender and medication status, and randomized by drawing numbers corresponding to ID numbers to either training or control group by a staff member not involved in training or testing. Staff members responsible for testing were not involved in conducting the training sessions for the intervention group, which took place at participants' schools by a teacher or other person designated by a school official. Training sessions took place at the participant's school during regular school hours and all testing took place at the Departments for Child and Adolescent Psychiatry in Vestfold or Telemark Hospital Trusts, Norway. The study was approved in advance (18.06.2009) by the Regional Committees for Medical and Health Research Ethics (REC South East: available on their webside: https://helseforskning.etikkom.no/ikbViewer/page/prosjekterirek/prosjektregister?_ikbLanguageCode=us&p_dim=34977&9F508B87E7D8620DE040F28156A418DC.p_search_id=26503.

This study is also registered as ISRCTN19133620 (www.controlled-trials.com).

Recruitment and enrollment began in 01.08.2009 and follow-up testing was completed 31.12.2010.

### PC-based training program

The WM training program for the treatment group consisted of 13 different PC-based exercises (CogMed). The same training program has been used in all of the computerized WM studies referred to in this study.

The training program includes three letter span tasks (all forward condition), three digit span tasks (one forward condition, two backward conditions), and seven visuospatial tasks (all forward sequenced), including static visuospatial tasks (one 2D visuospatial task, one 3D visuospatial task), and two dynamic visuospatial tasks, in which students recall the positions of rotated or moving objects. Nine of the tasks are presented purely in visual format, and four are delivered with an auditory input. Eleven of the tasks are forward sequenced, while only two are reverse-order tasks. A critical feature of the program is adaptivity, i.e. the level of difficulty is adjusted continuously to the individual student's skill level throughout the training program. The training group completed 10–15 trials of eight exercises each day for a total of 115 WM trials per day. Training time averaged about 30–40 minutes per day, depending on the exercise set and the student's performance level. Training participants agreed in advance with the person administering the training program on a reward schedule for achieving training targets. They received daily verbal and visual feedback about increases in performance and were rewarded after training by being allowed to play the RoboRacing-computer game. Every fifth day participants received an additional individualized reward agreed to by the school administration. Parents were instructed not to provide any rewards. Four subjects completed less than the planned number of 25 training sessions (3 had 24 sessions and one had 21). The training program has a built-in compliance measure, which is calculated by subtracting the Start Index (results of day 2 and 3) from the Max Index (results from the two best training days). Average improvement index in the training program for the whole group was 24 (s.d. 8), slightly less (23) for those completing less than 25 training days. Four subjects discontinued training for about one week or more during the planned training period due to a flu epidemic. These days were compensated for by training additional days when they returned to school. Due to varying lengths of sick leave, one participant trained for 26 days and three trained for 30 days.

### Neuropsychological measures

*Digit span* is a common neuropsychological test in the WISC-IV battery used to assess auditory working memory [34]. The test features forward and backward tasks that tap distinguishable cognitive processes [35], [36]. Digits Forward involves repeating a sequence of numbers in the same order right after they have been read aloud, and Digits Backward involves repeating the numbers in reverse order.

*The Leiter International Performance Scale-Revised (Leiter–R)* is a proven cognitive assessment tool that has a unique response format which is expressively nonverbal [37], [38]. Two tests from this battery were chosen to assess Visual WM: The Remembering Game (Visual span, forward), and The Backwards Game (Visual span, backward). In both tasks, the stimuli to be remembered are visual images of familiar items (e.g. a frog, a ship, a shoe).

In *the Letter-Number Sequencing task*, a list of intermingled numbers and letters of increasing lengths are read aloud [34]. Subjects are asked to recall the numbers in ascending order and the letters in alphabetical order, with numbers always coming first. Mental manipulation is one of the cognitive processes taxed by this task involving simultaneous reordering of a series of letters and numbers [39].

In *the Sentence Span task*, subjects must reproduce the words in a sentence read aloud in the exact same sequence [40], [41]. The sentence length is increased gradually until three incomplete sentences are registered. The manipulation component of WM is taxed in that rehearsal and updating is needed as the child's phonological memory span is surpassed [9].

### Composites

Raw scores were converted into scaled scores based on the norms provided in the respective manuals before aggregating the results into composites. The following composites were then calculated based on a component analysis deriving an empirically-based tri-partite division of WM aspects. Auditory WM: (*Digit span forward* + *Digit Span backward*)/2, Visual WM: (*Leiter R forward* + *Leiter R backward*)/2, and Manipulation WM (*Letter-Number* + *Sentence Span*)/2. A reliability analysis was performed for each of the composites indicating satisfactory internal consistency. The Cronbach alpha coefficient was .772 for the six items in the Visual WM composite, .747 for the six items in the Auditory WM composite and .720 for the six items in the Manipulation WM composite.

### Data analysis

Data analyses were conducted using the statistical package IBM SPSS Statistics for Windows, version 18.0 (SPSS, Inc., Chicago, IL). Demographic and clinical background information at baseline was analyzed with Analysis of Variance for continuous variables and Chi-square for categorical variables. Baseline levels for all dependent measures were compared between the un-medicated and the medicated groups.

The six tasks in the study taxing working memory components were subjected to a principal components analysis (PCA) with varimax rotation. The PCA revealed the presence of three pairs of components that grouped together (further description in Results section).

Baseline performance of the two groups and between medicated and unmedicated subjects were analyzed with Analysis of Variance (ANOVA).

Treatment effects were analyzed with Repeated Measures Analysis of Variance (Pre-test compared to Post-test 1 or Post-test 2) as within-subjects factor and group as the between-subjects factor. Only statistical measures of the interaction between time points and group are reported, as that accounts for a differential effect in one of the groups. Effect sizes are reported as Cohens *d*, computed as the difference between standard scores at Pre-test and Post-test 1 or Post-test 2 in the two groups, divided with the pooled standard deviation at baseline. The gain scores for all composites at Post-test 1 and Post-test 2 were calculated by subtracting the Pre-test baseline value from the Post-test 1 and Post-test 2 scores, which gave a net improvement score at Post-test 1 and Post-test 2. The net change on the Digit Forward task from Pre-test to Post-test 2 was included as a covariate in subsequent analyses of the Manipulation WM composite in order to control for the effect any increase in simple maintenance capacity might have had on the outcome.

## Results

The scree test showed a levelling off of Eigenvalues between 3 and 4 factors, but without a decisive elbow. The Eigenvalue of the third factor was .96, and the fourth factor .83. We decided to retain three factors explaining 69% of the variance. The results are presented in Table 2. The factors are numbered and given a title according to the subfunction we consider them to measure. The subfunctions will be discussed in the Discussion section.

**Table 2:** Factor structure of working memory measures derived from clinical sample (n = 67).

	I b Visual WM	II b Manipulation WM	III b Auditory WM
Digit Span, forward a	−.159	.291	**.804**
Digit Span, backward a	.473	−.164	**.662**
Leiter R, visual span, forward a	**.858**	.091	.152
Leiter R, visual°span, backward a	**.825**	.227	−.112
Letter-Number a	.121	**.771**	−.063
Sentence Span a	.118	**.676**	.278
Eigenvalue	2.00	1.17	0.96
% variance explained	33.28	19.46	15.94

a Raw score data registered at Pre-test.

b Extraction Method: Principal Component Analysis. Rotation Method: Varimax with Kaiser Normalization.

Factor 1 explained 33.3% of the variance and received salient loadings from the two Leiter tasks, Factor 2 explained 19.5% of the variance and received the high loadings from Letter-Number span and Sentence Span. Digit Span forward and Backward loaded on Factor 3, which explained 15.9% of the variance. Based on the principal component analyses, we derived the following composite scores: Visual Working Memory, Auditory Working Memory, and Manipulation Working Memory. A correlational analysis of the individual measures did not reveal any significant correlation between any of the composite scores, indicating that the tasks tapped separable aspects within the same construct.

Initially, a MANCOVA was conducted to test the overall changes caused by training. The values for the three composite measures both at T2 and T3 were entered and the baseline values at T1 were used as covariates to control for any minimal group differences. The MANCOVA showed an overall significant group effect, F =  (6,54) 3.73, *p* =  .004, Eta^2^ = .29. At the baseline level, the two groups differed with regard to the Auditory Working memory score. As evident from Table 3, the control group performed 1.0 scaled score better than the training group, F =  (1, 66) 7.24, *p* = .009, Eta^2^ = .10. There were no differences between the medicated and unmedicated participants on any of the outcome measures.

**Table 3:** Mean, standard deviation and ANOVA group comparisons.

	Pre-test (PreT)		Post-test 1 (PT1)		Post-test 2 (PT2)		PreT to PT1 comparison^a^			PT1 to PT2 comparison^a^			PreT to PT2 comparison^a^		
	Training group	Control group	Training group	Control group	Training group	Control group	*F*	*P*	*d* b	*F*	*p*	*d* b	*F*	*p*	*d* b
Visual WM	9.7 (2.6)	10.5 (2.1)	11.9 (2.7)	11.1 (1.7)	12.1 (2.8)	10.2 (2.5)	7.45	.01**	0.67	4.21	.05*	0.43	8.48	.01**	1.11
Auditory WM	8.0 (1.6)	9.0 (1.7)	8.8 (1.7)	8.8 (2.3)	8.5 (2.2)	8.7 (2.1)	7.65	.01**	0.66	0.52	ns	−0.19	3.62	.05*	0.47
Manipulation WM	6.7 (1.9)	7.0 (2.7)	8.4 (2.2)	8.1 (2.3)	8.6 (2.5)	7.2 (2.7)	1.59	ns	0.27	4.12	.05*	0.46	6.85	.05*	0.73

a Mixed between-within subjects analysis of variance – reported: interaction effect time x group (Wilk's Lambda).

b Cohens *d* calculated as the mean difference between groups divided by pooled standard deviation at baseline.

* *p*<.05, **p<.01.

**Figure 2:**
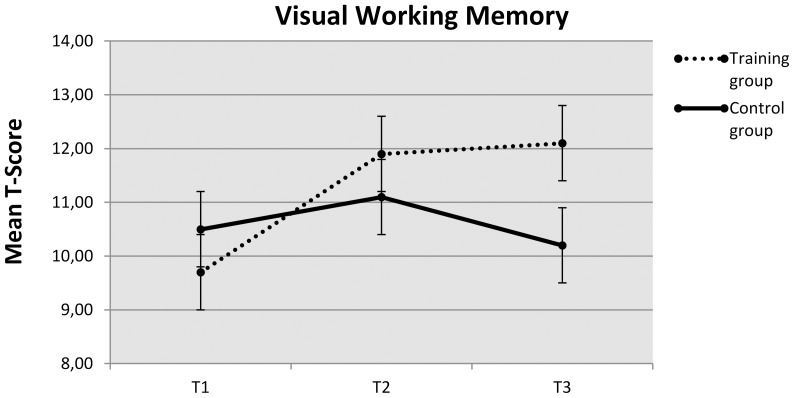
Mean t-score of Visual Working Memory composite at baseline (T1), after training period (T2) and 8-months later (T3) for Training and Control groups.

Table 3 presents the results of the repeated measures analysis of variance (ANOVA) conducted to assess the effect of WM training on the composites for Visual WM, Auditory WM and Manipulation WM. The composites for Visual WM and Auditory WM showed a significantly higher gain for the training group immediately after training compared to the control group, while the Manipulation WM composite did not. All of the composite scores, however, were significantly improved for the training group compared to the control group at testing eight months after training. The increase in scaled scores for the training group over the long term ranged from 0.5 for the Auditory WM composite and 1.9 for the Manipulation WM composite, to an increase of 2.4 for the Visual WM composite. Effect sizes measured in Cohens *d* over the long term ranged from .47 for the Auditory WM composite and .73 for the Manipulation WM composite, to 1.11 for the Visual WM composite. Whereas the Auditory WM composite showed a slight loss of training effect in the period extending right after the training period until the eight month follow-up testing, the gains achieved after the training period on the Visual WM and Manipulation WM composites persisted for the training group until the eight month follow-up testing. The change in performance on the Visual WM composite by the two groups over the long term is presented in Figure 2.

Repeated measures ANOVA were conducted on the gains registered for the training group alone on the Visual and Auditory WM composites to compare the relative change between the modalities. The analyses showed a significant gain in improvement on the Visual modality composite compared to the Auditory WM modality composite for the training group over the long term, (F (2, 30)  = 7.34, *p* =  <.01, partial Eta^2^ = .33). The same analysis was conducted for the control group to check if the results could be due to test-retest effects. No significant difference between the Visual and Auditory WM composites over the long term was found for the control group, (F (2,32)  = 2.09, *p* = ns, partial Eta^2^ = .12).

Analysis of covariance (ANCOVA) was conducted to examine the likely influence of an increase in simple storage capacity span on the Manipulation composite. Since both of the measures involving more complex manipulation (i.e. Letter-Number span and Sentence Span) were auditory, a measure presented in the same modality should be chosen to control for any increase in simple storage capacity. Both Digit span forward and backward loaded on the same factor in the principal component analyses, perhaps indicating that the manipulation load of Digit Span backward is low and not sufficient to override the effect of modality. However, the Digit Span backward task is considered to require additional manipulation demands compared to the Digit Span forward task [42]. Thus, we considered the Digit Span forward task to be a purer measure of simple auditory storage and the best candidate to control for any increase in simple storage capacity. Participants' increase in score on the Digit Span forward task from Post-test 1 to Post-test 2 was therefore used as a covariate in the analysis. After controlling for any increase in simple storage capacity, there was still a significant difference in the Manipulation WM measure for the training group compared to the control group over the long term, F (2, 60)  = 3.38, *p*< .05, partial Eta^2^ = .10.

All of the above analyses were computed excluding the five children who had changed medication during the project period. The background for omitting these 5 children was to avoid medication effects confounding the results. The consequence of removing these five children was to reduce the group effect size. The same analyses were conducted including the five children that changed medication, but this did not change any of the main findings.

## Discussion

The aim of the current study was to investigate the long-term near-transfer effects of a PC-based WM training program on standard measures of WM. The subjects were preadolescent children with ADHD, who promoters of the training program argue might benefit from a remedial non-medicinal intervention to treat impaired working memory functioning. The age group 10–12 was chosen as it is common for children diagnosed with ADHD in Norway to first come into contact with the specialized psychiatric services during these years as a consequence of increasing educational, social and self-regulatory demands, and the restricted age range would reduce age-related variance in the relevant variables. Most evidence also suggests that there is a stable, linear trajectory of WM development during these preadolescent years [43], suggesting that any improvement compared to controls would likely be a consequence of training and not of other developmental factors.

The first step was to form composites based on a principal component analysis of the six working memory tasks administered in the study. Results from the component analysis indicated grouping the measures into composites taxing Visual, Auditory and Manipulation aspects of WM. The first two are equivalent to what some authors refer to as Short Term Memory [30]. This grouping is consistent with the Domain-general model of WM that posits domain-specific storage components (in the visual and auditory domains), and a centralized component for manipulation, e.g. the central executive.

First, we expected there to be a significant improvement on all composite measures in the training group compared to the control group. Although this prediction may seem evident, not all WM training studies using the same program used in this study have shown consistent gains across tasks [12], nor across modalities [44]. The significant finding in favor of the training group on all composite measures in the current study over the long term provides evidence that adaptive training programs can be used to enhance performance on tasks measuring near-transfer effects. A similar finding was shown in another study using composite scores to assess gains after WM training [45]. An important issue, however, is whether a gain on a specific task is at all meaningful, given that the claim of promoters of such programs is a widespread effect on learning. We are indeed sceptical of any such ambitious claims, and the investigation of generalizable effects from the same study [26] reveal meager far-transfer effects. And yet, just as a golfer will train many individual skills in order to lower his or her handicap, the relationship between individual skills and lowering your handicap is not straight-forward. The gain in several aspects of WM by the training group in the current study was shown to persist on all measures compared to controls after eight months, which indicates that the training was having a lasting effect on something. As shown in Table 2, the training increased the absolute level of performance on the composites by from 0.5 to 2.4 scaled scores. Even so, it could be argued that these types of improvements are so mundane, that they might not justify the tremendous investments in time and resources involved in conducting such a training program.

Second, we predicted that the gain in performance by the training group from training on similar tasks would be relatively higher on a composite taxing Visual WM than the composite taxing Auditory WM due to the predominance of visually presented tasks in the training program. Of the 13 tasks in the PC-based training program, nine are presented purely in visual format, and only four have an additional auditory component. The finding of a significantly higher gain on the Visual WM composite compared to the Auditory WM composite for the training group in the current study supports the assumption that cognitive skills learned alike look alike. One implication is that the presentation form used in the training program may be a limiting factor in the transferability of learning to other domains. A spillover effect to the auditory modality for the training group is also evident, whereas no differential gain in either modality is registered for the control group. Importantly, enhancement through training may be of particular relevance for children with ADHD, who show highest performance gains after training relative to controls precisely on WM components most impaired in children with ADHD [1].

The third prediction relates to the manipulation component in the WM construct. Although there is general agreement among the leading WM theories of a “manipulation” component in WM [8], the exact nature and complexity of this cognitive process is still not clearly defined. In a study from 2001, Perry and co-authors (2001) support a separation of WM tests characterized as either transient “online” storage and retrieval tasks or executive-functioning WM tasks. The Engle-Shipstead team (2012) proposes a further distinction between simple and complex manipulation, considering backward versions of visual and auditory tasks as indications of simple manipulation capacity. The component analysis conducted on the six WM measures administered in the study indicated a third factor beyond forward and backward versions of common WM tasks, which we have labeled a Manipulation component. Interestingly, the effect of the training on the Manipulation composite for the training group after eight months was larger than the effect on the Auditory composite, despite the fact that both tasks in the Manipulation composite were delivered in an auditory format. This would seem to suggest that the gain was not due to improved auditory efficiency. Further, the improvement in the Manipulation composite over the long term seems to track closer to the improvement in the Visual composite than the Auditory composite. This suggests that the gain experienced by the training group involved an improvement in a process more closely related to the process underlying the gains on the Visual composite. Analyses controlling for the effect of an increase in simple maintenance capacity after training indicated that the improvement in Manipulation seems to be independent of any increase in simple maintenance capacity. An unexpected increase in performance in the control group from Pre-test to Post-test 1 seems to explain the lack of a significant difference in performance on this composite between the groups immediately after the training period. The fact that both groups showed gains, however, suggests that the participants were not being selectively influenced by the fact that the testers were not fully blind to participants' group assignment. The significant result for the training group on the Manipulation composite over the long term seems to be driven by the control group showing a sizeable regression from Post-test 1 to Post-test 2. This raises an intriguing possibility that an improvement in task specific skills from computerized training (e.g. Auditory or Visual WM) could be enhancing a sustained capacity for more complex tasks other than a simple test-retest effect. Future studies could investigate more systematically whether an increase in near-transfer tasks may transfer to measures of sustained attention or vigilance over the long term.

An important theoretical issue is whether the results support a model incorporating domain-general resources involving a central executive supplemented by domain-specific storage functions (e.g. phonological and visuospatial loops), or a model in which WM resources are separable across the verbal and visuospatial domains. First, the component analysis indicating a factor other than a Visual and Auditory component would seem to support the domain-general model. Second, the gain in the Manipulation composite for the training group persisted after controlling for a simple increase in storage capacity, suggesting the existence of a third factor we assumed to involve manipulation. Third, the gain from training as measured by effect size was almost identical right after training despite the imbalance in Visual and Auditory training tasks, suggesting an equivalent learning gain in both modalities. In the domain-specific model, performance on auditory and spatial WM tasks should not be highly associated [27]. On the other hand, the long-term effect of the gain in performance was quite different in the Visual and Auditory modalities. One interpretation of this disparity is that whereas short-term gains may involve fluctuations in simple storage capacity affecting the results, lasting change (i.e. learning) takes place primarily in a unified auditory or spatial system. The long-term results would seem to provide evidence in support of the domain-specific model, and underlines the importance of long-term testing when assessing the effectiveness of training interventions.

In interpreting the near-transfer gains, the question should be asked whether the gains could be explained by enhancement of some cognitive process other than an improvement in WM. A common assumption in studies on WM training is that improvement on WM tasks represents an improvement in an aspect of executive function localized in prefrontal structures. Based on research with patients with prefrontal lesions, however, researchers have shown that a person's ability to perform actions elicited by visual, situational cues remains intact compared to healthy controls despite serious impairment in prefrontal systems [46]. The question raised is thus whether the children in the training group in the current study could be improving systems governing automatic response patterns and not prefrontal structures normally associated with WM tasks. This would account for a task-specific improvement that would likely not be generalizable to other cognitive domains. Alternative explanations will have to be addressed in future studies. The current study has at least shown that preadolescents with ADHD are able to significantly improve task-specific skills lasting at least 8 months through systematic training, which is evidence supporting the viability of targeted training interventions for this clinical group. Whether the gain in performance on WM measures represents an improvement in other cognitive domains or to everyday functioning is addressed separately [26].

Limitations in the current study include not having a normal control group with which to compare performance gains. The aim of the study, however, is not to boost performance beyond a normal level, but to see if children with ADHD, who as a group suffer from impaired WM function, can improve their performance on similar tasks through training. Some authors have suggested experimental designs comparing computerized WM training with other interventions such as inhibition training. Although this is a possibility, it would introduce a new range of variables potentially influencing the results. An important limitation is that even though the testers were not informed of participants' group assignment, they were in reality not blind to group assignment. Another important consideration is that the narrow focus on near-transfer effects of the training program in this study could be viewed by some as a limitation, because any gains could be misinterpreted to refer to generalizable WM gains. Unless training gains transfer by enhancing other useful skills, it could be argued that the extensive resources involved in such a training program should be allocated elsewhere. We agree with this highly relevant cost-benefit consideration. However, we maintain that narrow, near-transfer results from training programs should also be investigated and reported as they can shed light on the effectiveness of task-specific interventions for specific clinical groups and may be informative for the theoretical debate on the WM construct and components.

## Conclusion

The five-week, computerized training program significantly enhanced performance on similar tasks for the training group compared to the control group. The increase in Visual WM performance was larger over the long term compared to the increase in Auditory WM performance, suggesting that the presentation format of the training exercises is an important variable affecting learning. The gain in performance on the Manipulation composite over the long term was beyond any increase due to heightened simple storage capacity. The results are interpreted as evidence supporting aspects of both the domain-general and domain-specific models of working memory. Alternative explanations for the gain in WM scores are suggested and should be investigated in future studies.

## Supporting Information

Checklist S1 **Consort Checklist of information to include when reporting a randomised trial.**
(DOC)

Protocol S1 **Trial study protocol- English translation.**
(DOC)

Protocol S2 **Trial study protocol- Norwegian original.**
(DOC)
